# Immunopathogenesis of Immune Checkpoint Inhibitor-Related Adverse Events: Roles of the Intestinal Microbiome and Th17 Cells

**DOI:** 10.3389/fimmu.2019.02254

**Published:** 2019-09-26

**Authors:** Ronald Anderson, Annette J. Theron, Bernardo L. Rapoport

**Affiliations:** Department of Immunology, Faculty of Health Sciences, University of Pretoria, Pretoria, South Africa

**Keywords:** adenosine 5-triphosphate, cytotoxic T-lymphocyte-associated protein (CTLA-4), ipilimumab, immune-related adverse events (IRAEs), interleukin-17, microbiome, nivolumab, programmed cell death protein-1 (PD-1)

## Abstract

The advent of novel, innovative, and effective anti-cancer immunotherapies has engendered an era of renewed optimism among cancer specialists and their patients. Foremost among these successful immunotherapies are monoclonal antibodies (MAbs) which target immune checkpoint inhibitor (ICI) molecules, most prominently cytotoxic T-lymphocyte-associated protein (CTLA-4) and programmed cell death protein-1 (PD-1) and its major ligand, PD-L1. These immunotherapeutic agents are, however, often associated with the occurrence of immune-mediated toxicities known as immune-related adverse events (IRAEs). The incidence of severe toxicities increases substantially when these agents are used together, particularly with CTLA-4 in combination with PD-1 or PD-L1 antagonists. Accordingly, dissociating the beneficial anti-tumor therapeutic activity of these agents from the emergence of IRAEs represents a significant challenge to attaining the optimum efficacy of ICI-targeted immunotherapy of cancer. This situation is compounded by an increasing awareness, possibly unsurprising, that both the beneficial and harmful effects of ICI-targeted therapies appear to result from an over-reactive immune system. Nevertheless, this challenge may not be insurmountable. This contention is based on acquisition of recent insights into the role of the gut microbiome and its products as determinants of the efficacy of ICI-targeted immunotherapy, as well as an increasing realization of the enigmatic involvement of Th17 cells in both anti-tumor activity and the pathogenesis of some types of IRAEs. Evidence linking the beneficial and harmful activities of ICI-targeted immunotherapy, recent mechanistic insights focusing on the gut microbiome and Th17 cells, as well as strategies to attenuate IRAEs in the setting of retention of therapeutic activity, therefore represent the major thrusts of this review.

## Introduction

The advent of monoclonal antibody (MAb)-based, immune checkpoint inhibitor (ICI)-targeted immunotherapy has dramatically and successfully transformed the landscape of the treatment of several types of advanced malignancies, particularly non-small cell lung carcinoma (NSCLC) and melanoma (1, 2). To date, this type of immunotherapy is based almost exclusively on the restoration of anti-tumor immunity, resulting from the blockade of one or both of two distinct types of ICI proteins, *viz.*, cytotoxic T-lymphocyte-associated protein-4 (CTLA-4; also known as CD152) (3) and programmed cell death protein-1 (PD-1; CD279) (4), which affect different stages and mechanisms of effector T cell activation (5, 6).

While clearly representing a watershed in the immunotherapy of cancer, the full therapeutic potential of MAb-based ICI-targeted immunotherapy of advanced cancer remains to be realized. This is due to the accompanying risk of development of a spectrum of unusual, immunotherapy-related, potentially harmful, immunological reactions known as immune-related adverse events (IRAEs). These reactions occur with surprisingly high frequencies of around 30, 50, and 70–90% in patients treated with individual PD-1 and CTLA-4 antagonists or with combinations of these, respectively, and may restrict the duration and efficacy of immunotherapy (1, 2). All organ systems are vulnerable, especially the skin, gastrointestinal system, liver and endocrine system, while the reactions range from mild to even fatal (1, 2).

Currently, relatively little is known about the immunopathogenesis of MAb-based ICI therapy-associated development of IRAEs. This represents a significant gap in knowledge, which if successfully addressed may enhance the anti-cancer efficacy of this type of immunotherapy. Accordingly, the current review is focused on the following recent insights, which may contribute to attainment of this goal: (i) pre-existing autoimmune disease and eligibility for ICI-targeted immunotherapy; (ii) relationships between immune restoration and development of IRAEs; (iii) composition of the gut microbiota as a determinant of the efficacy of ICI-targeted immunotherapy; (iv) mechanisms underpinning ICI-MAb-mediated immune restoration and development of IRAEs; (v) role of adenosine 5′-triphosphate (ATP) derived from gut commensal bacteria in promoting differentiation of Th17 cells; (vi) the involvement of intestinal Th17 cells in the pathogenesis of some types of IRAEs and restoration of anti-tumor immunity; and (vii) a consideration of potential immune-based therapies to ameliorate development of IRAEs. These sections are preceded firstly, by a brief outline of the immunosuppressive activities of CTLA-4 and PD-1, and, secondly, the chronology of United States (US) Food and Drug Administration (FDA) approval, as well as initial therapeutic applications, of several of the earlier, prominent ICI-targeted MAbs.

## CTLA-4 and PD-1 Immune Checkpoint Inhibitors

CTLA-4 is expressed constitutively on regulatory T cells (Tregs) (5, 6). In the context of an efficiently functioning immune system, Tregs are critically involved in maintaining a level of immunological homeostasis necessary to prevent development of autoimmune and auto-inflammatory diseases. If overactive, however, excessive Treg-mediated immunosuppression may result in attenuation of both anti-tumor and anti-infective host defenses (5, 6). Depending on the subtype of Tregs, immunosuppressive activity is achieved by several mechanisms (7). These include direct interactions of CTLA-4 with the co-stimulatory, counter-receptors, B7-1 (CD80) and B7-2 (CD86), expressed on antigen-presenting cells (APCs), predominantly activated dendritic cells (DCs), as well as macrophages. This results in suppression of the antigen-presenting and other T-cell-activating functions of these cells (5, 6). In addition, CTLA-4 expressed on Tregs can also enable capture, sequestration, and degradation of B7-1 and B7-2 expressed on DCs by a process of trans-endocytosis, which also results in attenuation of the co-stimulatory activities of APCs (6, 8). Other mechanisms include: (i) production of the broad-spectrum, immunosuppressive cytokines, transforming growth factor-β1 (TGF-β1) and interleukin (IL)-10 (5, 6); (ii) depletion of antigenic peptide/MHC class II complexes on the surface of DCs (9); and (iii) IL-10-mediated induction of alternative, anti-inflammatory M2-like macrophages, resulting in decreased activation of effector T cells (10). M2-like macrophages, largely through production of TGF-β1, also potentiate the formation of Tregs. This is achieved firstly, by protecting these cells from apoptosis during thymic development, and, secondly, by inducing the transition of immature Tregs into functionally mature cells (11, 12).

Notwithstanding inducing over-activity of Tregs, several other mechanisms exist by which CTLA-4 contributes to suppression of anti-tumor immunity. Most importantly, these include expression and release of CTLA-4 by tumor cells *per se* (13–15), as well as release of microvesicle-packaged CTLA-4 by mature myeloid DCs in the tumor microenvironment (16, 17). Both of these mechanisms target tumor-infiltrating T cells (TILs), contributing to an ongoing cycle of sustained immunosuppression.

Unlike Tregs, surface expression of CTLA-4 by both naïve and anti-tumor CD4+ and CD8+ effector memory T cells only occurs following major histocompatibility complex (MHC)-dependent activation of these cells by APCs. This happens as a result of engagement of the T cell receptor (TCR) for specific antigen in the setting of generation of co-stimulatory signals, resulting from the interaction of CD28 (the IL-2-inducing counterpart of CTLA-4) expressed on T cells with B7-1/B7-2 on APCs (5, 6).

Like CTLA-4, PD-1 is also a member of the B7/CD28 family, but in contrast to CTLA-4, PD-1 and its ligands, PD-L1 (CD274) and PD-L2 (CD273), the former having the highest affinity for PD-1, are more broadly expressed than the CTLA-4/B7 axis. In this context, PD-1 is expressed not only by activated T cells, but also by B cells and cells of the myeloid lineage (5). The ligand, PD-L1, is expressed on various types of immune and non-immune cells, including tumor cells, while PD-L2 is predominantly expressed on APCs (5). PD-1-mediated suppression of tumor-targeted immune mechanisms involves the interaction of this ICI expressed on activated, anti-tumor CD4+ and CD8+ effector T cells with PD-L1 expressed on tumor cells. Unlike CTLA-4, which suppresses the initial priming events in T cell activation, engagement of PD-1 inhibits the effector phase, resulting in the failure of both T cell proliferation and production of the immunopotentiating cytokines, IL-2, tumor necrosis factor-α (TNF-α) and interferon (IFN)-γ, while also driving a pro-apoptotic state (5). In addition, PD-L1-expressing DCs may also drive the progression of naïve, PD-1-expressing CD4 Tregs to the mature, immunosuppressive phenotype, favoring co-operative impairment of anti-tumor host defenses due to co-expression of CTLA-4 and PD-1 by Tregs (5, 18, 19). As mentioned in a later section of this review, these highly efficient Tregs appear to drive intestinal immunosuppression via mechanisms involving hydrolysis of adenosine-5'-triphosphate (ADP) derived from commensal microorganisms (20–22).

The aforementioned immunosuppressive activities of CTLA-4 and PD-1 are summarized in Table 1.

**Table 1:** Immunosuppressive activities of CTLA-4 and PD-L1.

**Immune checkpoint inhibitor**	**Cell type**	**Immunosuppressive activity**	**References**
CTLA-4	Tregs	Interaction with CD80 and CD86 on APCs resulting in decreased antigen presentation and T cell activation	(5, 6, 8)
CTLA-4	Tregs	Production of broadly immunosuppressive TGF-β1 and IL-10 resulting in induction of M2 macrophages which further potentiate formation of Tregs	(5, 6, 10–12)
CTLA-4	Tregs	Depletion of antigenic peptide/MHCII complexes on DCs	(9)
CTLA-4	Tregs	Depletion of T cell-activating ATP and formation of immunosuppressive adenosine via co-expression of CD39 and CD73	(20–22)
CTLA-4	Tumor cells	Decreased antigen presentation and T cell activation	(13–15)
CTLA-4	Mature myeloid DCs	Release of vesicle-packaged CTLA-4 in the tumor micro-environment	(16, 17)
PD-1	CD4^+^ and CD8^+^ effector T cells	Interference with tumor-targeted immune mechanisms via binding with PD-L1 expressed on tumor cells	(5)
PD-1	DCs	Interaction with PD-1 on immature Tregs promotes transition to the mature, CTLA-4/PD-1, immunosuppressive phenotype	(5, 18, 19)

*APC, antigen presenting cell; ATP, adenosine 5′-triphosphate; DC, dendritic cell; MHC, major histocompatibility complex; Treg, regulatory T cell*.

## Immune-checkpoint Inhibitor-Targeted MAbs

The first MAb to receive approval by the US FDA for clinical application in the setting of selected, advanced malignancies was the CTLA-4-targeted agent, ipilimumab, a fully human IgG1 MAb, approved initially for treatment of advanced melanoma in 2011 (23, 24). A list of more recently approved ICI MAbs is summarized in the Supplementary Table with supporting references.

To date, the major beneficiaries of MAb-based ICI therapy are patients with advanced melanoma (ipilimumab alone or with a PD-1 inhibitor) and NSCLC (PD-1/ PD-L1), with these malignancies appearing to be particularly responsive to this type of immunotherapy and associated with durable responses in ~25% of patients (25, 26). In this context, it is noteworthy that single-agent administration of pembrolizumab (PD-1 antagonist) is now recommended by the US FDA and European Medicines Agency for the treatment of patients with advanced NSCLC who have a PD-L1 tumor promotion score (TPS) of ≥50% (27). Even more recently, based on the findings of the KEYNOTE-42 study, Mok et al. proposed that the TPS recommendation in respect of first-line administration of pembrolizumab in NSCLC be revised to include patients with even lower TPS values (28), however caution in this regard has been advocated by others who contend that patients with a lower TPS score should be treated with ICIs and chemotherapy (27).

## Types of Immune-Related Adverse Events (IRAEs)

IRAEs associated with ICI therapy include those of dermatological, gastrointestinal (GIT), pulmonary, hepatic, endocrine and ocular origin, as well as less frequent immune-toxicities such as type 1 diabetes mellitus and those of cardiac, neurological, and hematological origin. Dermatological toxicities can appear following the first dose of ICIs and can be ongoing. These skin rashes are most frequently maculopapular and mild in nature (29). Generalized pruritus and skin rash are seen more frequently with CTLA-4-targeted therapy compared to anti-PD-1/PDL-1-based therapy (30). In some instances, serious skin reactions such as Stevens-Johnson syndrome and toxic epidermal necrolysis have been described (31). Vitiligo has also been described in a small proportion of patients undergoing treatment with ICIs and interestingly this condition is associated with clinical benefit and long-term survival (32).

GIT side-effects in the form of mucositis, aphthous ulcers, gastritis, colitis, and abdominal pain may occur and the presence of diarrhea, with blood or mucus in the stool, can be seen. Severe complications can progress to toxic megacolon and perforation and must be ruled out in patients showing symptoms of peritonitis (33). In severe cases, infectious causes of diarrhea, including *Clostridium difficile* infection may be evident (33).

Pneumonitis is a serious IRAE reported in patients undergoing treatment with ICIs. Pneumonitis is more common with PD-1 and PDL-1 blockers, however the incidence is <1% and presents later during the treatment phase (34). Clinicians should typically be aware of the development of immune-related pneumonitis in a patient undergoing ICI-based therapy who experiences new symptoms of dyspnea and/or cough. If not managed promptly, this complication may be fatal (34).

Endocrine-associated IRAE symptoms are generally non-specific and include fatigue, mental state changes, headaches, and dizziness related to hypotension (35). Hypothyroidism is the most commonly documented endocrine abnormality, with Addison's disease and hypophysitis also having been reported (35). Clinicians should screen patients for thyroid function abnormalities, including performance of baseline thyroid function tests. Other hormonal evaluations may be indicated in some patients. Ophthalmological IRAEs in the form of episcleritis, uveitis or conjunctivitis have also been described. These abnormalities may be mild, moderate or severe (36). Neurological IRAEs include posterior reversible encephalopathy syndrome, aseptic meningitis, enteric neuropathy, transverse myelitis, and Guillain-Barre syndrome (37), as well as relapses of multiple sclerosis associated with rapid neurologic progression and death (38).

Infrequently encountered IRAEs include red cell aplasia (39), neutropenia (39), acquired hemophilia A (39), thrombocytopenia (39), hemolytic-uremic syndrome (39), pancreatitis (40), asymptomatic increases in serum amylase and lipase (40), renal insufficiency with nephritis (41), arthritis and sicca syndrome (42), myocarditis (43), and sarcoid-like syndrome (44).

These various types of IRAE are summarized in Table 2.

**Table 2:** Summary of organ specific immune-related adverse events associated with ICI treatment^*^.

**Dermatological**
• Pruritus
• Skin rash
• Stevens-Johnson syndrome
• Toxic epidermal necrolysis
• Dermatitis exfoliative
• Erythema multiforme
• Alopecia
• Vitiligo
**Gastrointestinal**
• Mucositis
• Diarrhea
• Aphthous ulcers
• Gastritis
• Colitis
• Necrotizing colitis
• Toxic megacolon
• Perforation
• Peritonitis
• Pancreatitis
**Hepatic**
• Immune-mediated hepatitis
**Pulmonary**
• Pneumonitis
• Sarcoid-like syndrome
• Interstitial lung disease
• Acute interstitial pneumonitis
**Renal**
• Nephritis, autoimmune
• Renal failure
**Endocrine**
• Hyperthyroidism
• Hypothyroidism
• Adrenal insufficiency
• Hypophysitis
• Type 1 diabetes mellitus
**Ophthalmological**
• Episcleritis
• Uveitis
• Conjunctivitis
**Cardiac**
• Myocarditis
**Neurological**
• PRES (posterior reversible encephalopathy syndrome)
• Aseptic meningitis
• Transverse myelitis
• Guillain-Barre syndrome
• Autoimmune neuropathy
• Demyelinating polyneuropathy
• Guillain-Barre
• Myasthenia gravis– like syndrome
**Hematological**
• Red cell aplasia
• Neutropenia
• Acquired hemophilia A
• Thrombocytopenia
• Hemolytic-uremic syndrome
**Rheumatological**
• Inflammatory arthritis
• Sicca syndrome

* *IRAEs have been described in the settings of both registered and investigational CTLA-4 and PD-1-/PD-L1-targeted MABs. Hypophysitis and colitis are most commonly, but not exclusively, associated with ipilimumab, and pneumonitis with PD-1 inhibitors. The incidence and severity of IRAEs increases significantly when CTLA-4- and PD-1/PD-L1-targeted MAbs are used in combination*.

## Immune-Related Adverse Events (IRAEs) and Pre-existing Autoimmune Disease

Given the key roles played by CTLA-4 and PD-1 in the maintenance of immunological homeostasis, it is not entirely surprising that intravenous, as opposed to direct intra-tumoral, administration of MAbs that target these ICIs, could pose the potential risk of generalized immune system over-reactivity and immune-mediated toxicities. Because of the prevailing belief that patients with advanced cancer and co-existent autoimmune disorders were at highest risk for development of severe IRAEs, such patients were often excluded from access to immunotherapy-based, anti-cancer clinical trials (45). This strategy has, however, been challenged by the findings of a number of retrospective studies recently reviewed by Johnson et al. (45) and Khan and Gerber (46). The former authors state that “autoimmunity is often exacerbated by ICI therapy, but is generally manageable with standard treatment algorithms and close multidisciplinary monitoring. Further, the activity of these agents appears to be comparable to that seen in unselected patients” (45).

This contention is supported by the findings of a study undertaken by Cortellini et al. (47). This retrospective study, which covered the period September 2013–May 2018, encompassed fifteen Italian centers and a total of 751 patients with various types of cancer, predominantly NSCLC (65.5%), melanoma (21.2%) and renal cancer (12.5%), treated with PD-1 (pembrolizumab or nivolumab)-targeted immunotherapy (47). Of these patients (male: female participants = 499/252), 85 (11.3%; 48 male, 37 female) had pre-existing autoimmune diseases (predominantly thyroid, dermatologic, and rheumatologic disorders), of which 17.6% of patients had clinically-active disease (47). Following immunotherapy, the incidence of IRAEs of any grade was found to be significantly higher when comparing patients with and without autoimmune diseases (65.9 vs. 39.9%). Multivariate analysis revealed significant associations of IRAEs of any grade with both clinically-active and –quiescent autoimmune disease (*P* = 0.162 and *P* = 0.005, respectively), as well as with female gender (*P* = 0.0004) and Eastern Cooperative Oncology Group Performance Status of <2 (*P* = 0.0030). However, no significant differences were detected between the groups of patients with and without autoimmune disorders with respect to the frequencies of grades 3–4 IRAEs, as well as with progression-free survival (PFS) and overall survival (OS) (47). On the basis of these findings, the authors contend that cancer patients with pre-existing autoimmune diseases need not be excluded from access to immunotherapy, albeit conditional on multidisciplinary evaluation and recommendation (47). In addition, the authors note that “the finding of a greater incidence of IRAEs among female patients ranks among the hot topics in gender-related differences in immune-oncology” (47).

In cancer patients with preexisting autoimmune disorders, including type 1 diabetes mellitus, the use of PD-1 and PDL-1 ICI antibodies, in particular, is frequently associated with development of mild IRAEs, which are usually easily managed and do not require treatment discontinuation, with a significant proportion of patients attaining clinical responses (48–51). Although current data supports the use of ICI-based therapy in patients with pre-existing autoimmune disorders or type 1 diabetes mellitus, the level of evidence in respect of this recommendation is low. In this context, it is noteworthy that cancer patients with pre-existing autoimmune disorders or type 1 diabetes mellitus were excluded from all prospective registration trials and only retrospective data is currently available in these clinical settings.

Accordingly, these findings should be confirmed in well-designed prospective, randomized studies with adequate end-points. Additionally, “real world” studies may show that the incidence of IRAEs is significantly higher than in the clinical trial setting.

## Association Between Immune Restoration and Development of IRAEs

In yet another somewhat surprising development, a number of recent studies has suggested that the occurrence of clinically manageable IRAEs may even be predictive of an effective, immunotherapy-induced anti-tumor immune response. Five of these studies, all retrospective analyses, mostly encompassing patients with advanced NSCLC, analyzed the associations between administration of anti-PD-1 targeted immunotherapy (nivolumab or pembrolizumab), development of IRAEs and treatment efficacy (52–56). The numbers of patients recruited to these studies varied from 38–559 and the occurrence of IRAEs from 36.8 to 63.0%, predominantly of grade I/II severity, with frequencies of grade III/IV severity reported in 2/43 (52), 1/70 (53), 1/38 (54), 15/106 (55), and 50/559 (56) patients. In all five studies, the duration of PFS was found to be significantly longer (*P* = 0.016–*P* < 0.0001) and objective response rates (ORRs) greater (*P* = 0.17–*P* < 0.0001) in patients who developed IRAEs (52–56). However, no clear associations between therapeutic efficacy and severity of IRAEs were evident, possibly reflecting the small numbers of patients in the majority of the studies.

Several other recently published retrospective analyses, encompassing patients with various types of advanced cancer, have reported similar associations between development of IRAEs and efficacy of both CTLA-4- and PD-1-directed immunotherapy. In this context, Shafqat et al. reported that 45/157 (28.7%) patients with various types of cancer who were treated with anti-PD-1/PD-L1 MAbs developed IRAEs, predominantly of endocrine origin, 21 of whom were treated with systemic corticosteroids (57). Interestingly, Cox regression analysis revealed that patients who developed IRAEs had a lower risk for disease progression or death [hazard ratio (HR) = 0.34; *P* < 0.001)], the median PFS rates being 4.2 and 24.4 months for those patients without and with IRAEs (57). Patients who received corticosteroids also had a significantly improved PFS (HR = 0.38; *P* = 0.03). The authors concluded that development of an IRAE is indicative of an immune system that is responsive to ICI-targeted immunotherapy (57).

A second study was based on a retrospective review of the medical records of 290 patients with different types of advanced cancer who received various types of immunotherapy (ICI MAbs, *n* = 64; cytokine therapies, *n* = 87; cancer vaccines, *n* = 53; combined therapies, *n* = 86, which were ICI MAb-based in 63 patients) (58). The types of ICI-targeted MAbs, cytokine-based therapies and cancer vaccines were not, however, mentioned. Ninety eight patients (34%) developed IRAEs, predominantly dermatitis (*n* = 57); 15 of these were graded as 3/4 IRAEs, 5 of which were treated with systemic corticosteroids. These severe IRAEs were detected in 6, 5, 3, and 1 patients who received ICI-targeted, cytokine-based, vaccine-based, and combination therapies, respectively. Relative to the other patients (*n* = 275), those who developed grade 3/4 IRAEs had an improved ORR (25 vs. 6%; *P* = 0.034) and disease control rate (DCR, 67 vs. 21%; *P* = 0.001), a longer median time to progression of disease (30 vs. 10 weeks; *P* = 0.004) and a longer median OS (15 vs. 8 months; *P* = 0.10). Of the patients who received corticosteroids, 4/5 continued to respond (58). These findings appear to indicate that the intensity of an IRAE may reflect the magnitude of immune restoration.

Two studies, also from the MD Anderson Cancer Center, one focused on patients with different types of advanced cancer and the other specifically on melanoma, have also highlighted the association between development of IRAEs and efficacy of ICI-targeted immunotherapy (59, 60). The first of these involved a retrospective review of records of patients (*n* = 427) with various types of advanced cancer who had received treatment with ICI MAbs, individually (ipilimumab, pembrolizumab, nivolumab, atezolizumab) or in combination (ipilimumab + nivolumab), during the period 2011–2017 (59). Of these patients, 202 (47.3%) developed one (*n* = 126) or more (*n* = 76) IRAEs, predominantly colitis/diarrhea. The authors reported that irrespective of immunosuppressive therapy, patients who developed IRAEs had significantly (*P* < 0.01) increased duration of OS relative to those who did not experience IRAEs. This relationship was most evident in patients who experienced three or more IRAEs in comparison with those who experienced two or less (*P* = 0.010) (59). In addition to concluding that “IRAEs are associated with favorable overall survival regardless of immunosuppression,” the authors also contend that “IRAEs involving multiple organs appeared to be beneficial for overall survival” (59).

The second retrospective analysis reported by these investigators covered the period 01/2010–04/2018 and was focused on ICI-induced colitis as a predictor of survival in metastatic melanoma (60). The study involved 346 patients recruited from a total of 1,983 who had previously received either CTLA-4-(*n* = 848) or PD-1/PD-L1-(*n* = 1,135)-based therapies (60). Of these, 173 (8.7%) who had developed immunotherapy-related colitis were matched with an equal number of control patients without colitis to receive immunotherapy with CTLA-4- or PD-1/PD-L1-targeted MAbs individually or in combination (60). Again, multivariate Cox regression analysis revealed that any grade of GIT IRAE was associated with a significantly improved OS (HR = 0.53; *P* < 0.01), as well as PFS duration (HR = 0.56; *P* < 0.01), which was unaffected by immunosuppressive therapy (60).

Similar findings to those described in the preceding reports (52–60) have also been reported by Maher et al. who conducted an analysis of seven studies, encompassing patients (*n* = 1,747) with metastatic or locally advanced urothelial cancer deemed suitable for immunotherapy with anti-PD-1/PD-L1 MAbs (61). The analysis was focused on the relationship between OS and development of “related adverse events of special interest (AESIs)” as well as “related immune-mediated adverse events” (imAEs). The frequencies of AESIs were 64 and 34% in responders and non-responders to immunotherapy, respectively, while the corresponding frequencies for imAEs were 28 and 12%. Development of AESIs was significantly associated with increased OS (HR = 0.45; 95% CI: 0.39–0.52) (61).

A *post hoc* analysis of a prospective study (the EORTC 1325/KEYNOTE-054 study) addressing the issue of the relationship between IRAEs and outcome of ICI-targeted immunotherapy of advanced melanoma, has been communicated recently (62). This is a double-blind, placebo-controlled trial focused on the efficacy (recurrence-free survival, RFS) of pembrolizumab in high-risk, stage III melanoma patients (*n* = 1,011, with 511 and 502 in the pembrolizomumab-treated and placebo groups, respectively) (62). The authors of this study detected a significantly longer RFS in the pembrolizumab-treated group relative to the placebo, control group (HR = 0.61; 95% CI: 0.39–0.95; *P* = 0.03). This was most notable after the onset of an IRAE (HR = 0.37; 95% CI: 0.24–0.57; *P* = 0.028), with frequencies of IRAEs in the pembrolizumab-treated and placebo control groups of 37.3 and 9%, respectively (62).

The authors of all of the above reports, which are summarized in Table 3, contend that the development of IRAEs, seemingly manageable in the majority of cases, is indicative of an immune system which is likely to be responsive to restoration of anti-tumor immunity following administration of ICI-targeted MAbs (52–60). With the exception of the study by Eggermont et al. (62), these studies are, however, retrospective in nature, involving different patient populations, with small numbers in some instances, different tumor types in the same study, and different clinical end-points. Consequently, these are of a low level of evidence, requiring confirmation in properly designed, adequately powered clinical trials with appropriate end-points.

**Table 3:** Summary of retrospective studies (*n* = 10) and one *post hoc* analysis of a prospective study describing positive associations between the efficacy of immune checkpoint inhibitor-targeted immunotherapy and development of immune-related adverse events (IRAEs).

**Tumor type**	^*^Study type	**Type of immune checkpoint-targeted immunotherapy**	**Frequency of IRAEs**	^**^Clinical benefit parameter (median)		**References**
				**OS**	**PFS**	
Advanced NSCLC (43 patients)	R	PD-1-targeted (nivolumab)	44%	^***^NR	^#^6.4 vs. 1.5 months (*P* = 0.01)	(52)
Advanced NSCLC (70 patients)	R	PD-1-targeted (nivolumab)	40%	NR	^+^12 vs. 3.6 months (*P* value stated as significant)	(53)
Advanced NSCLC (38 patients)	R	PD-1-targeted (nivolumab)	28.9%	NR	^+^91 vs. 41 days (*P* <0.001)	(54)
Advanced NSCLC (559 patients)	R	PD-1-targeted (nivolumab/pembrolizumab)	41.3%	20.5 vs. 8.5 months (*P*< 0.0001)	10.1 vs. 4.1 months (*P <* 0.0001)	(56)
Various types of malignancy, mostly NSCLC (106 patients, 77 with NSCLC)	R	PD-1-targeted (nivolumab/pembrolizumab)	37.7%	NR	10 vs. 3 months (*P* = 0.016)	(55)
Various types of advanced cancer (157 patients)	R	PD-1/PD-L1-targeted (nivolumab, pembrolizumab, atezolizumab)	28.7%	NR	24.4 vs. 4.2 months (*P* < 0.001)	(57)
Various types of advanced cancer (290 patients)	R	Various types, including immune checkpoint inhibitors only (*n* = 64) or combined with other types of immunotherapy (*n* = 63)	34%	15 vs. 8 months (*P* < 0.10)	30 vs. 10 weeks (*P* < 0.004)	(58)
Various types of advanced cancer (427 patients)	R	CTLA-4- and PD-1/PD-L1-targeted immunotherapy individually (ipilimumab, atezolizumab, nivolumab, pembrolizumab), or in combination (ipilimumab+nivolumab)	47.3%	Significantly improved (*P* < 0.001)	NR	(59)
Metastatic melanoma (346 patients; 173 with immunotherapy-related colitis identified from a cohort of 1,983 patients matched with 173 control patients)	R	CTLA-4- and PD-1/PD-L1-targeted immunotherapy individually and in combination	8.7% in the original patient cohort of 1,983	Significantly improved(*P* < 0.01)	Significantly improved (*P* < 0.01)	(60)
Metastatic or locally advanced urothelial cancer (an analysis of 7 studies encompassing 1,747 patients)	R	PD-1/PD-L1-targeted immunotherapy	“Adverse events of special interest (AESIs)” reported in 64% and 34% of responders and non-responders, respectively	Increased (HR: 0.45; 95%CI: 0.39–0.52)	NR	(61)
A *post hoc* analysis of a study involving high-risk stage III melanoma patients (*n* = 1,011) recruited to a prospective, double-blind, placebo-controlled trial with 511 and 502 patients in the immunotherapy and placebo groups, respectively	P	PD-1-targeted immunotherapy (nivolumab)	37.3 and 9% in the nivolumab-treated and placebo groups, respectively	Significantly improved recurrence free survival in those who experienced immunotherapy-related IRAEs (*P* = 0.028)		(62)

* *R, retrospective study; P, prospective study*.

# *IRAEs vs. IRAE-free groups*.

** *OS, overall survival; PFS, progression-free survival*.

*** *NR, not reported*.

+ *Upper limit of confidence interval not reached for the group of patients who experienced IRAEs*.

It is also noteworthy that IRAEs have been associated with sustained clinical benefit in patients requiring treatment discontinuation due to these toxicities. The memory element of this immune response appears to be associated with a possible persistent, clinical benefit even following discontinuation of treatment. Intriguingly, this effect could be observed even if a complete response is not attained in these patients (63). However, it is essential to note that although the development IRAEs is associated with clinical benefit in terms of OS, some of these “off-target” systemic effects may be persistent, or even irreversible, compromising the quality of life of these patients.

The aforementioned findings seemingly indicate that the critical requirements for achieving effective, ICI-targeted, anti-tumor immunotherapy include not only raising the magnitude of immune restoration to levels necessary to overcome the impediment of existing immunosuppression, but also galvanizing the immune system into apparent overdrive as evidenced by the frequency of IRAEs affecting various organs.

## C-reactive Protein and Interleukin-6 as Predictors of Both the Efficacy of Immune Checkpoint Inhibitor-Based Therapy and Development of IRAEs

There is considerable current interest in the identification of systemic biomarkers which are predictive of the efficacy of ICI-targeted immunotherapy, as well as susceptibility for development of IRAEs. In this context, several studies have reported on the apparent utility of measurement of serum C-reactive protein (CRP), a surrogate for IL-6, as a strategy to predict responsiveness of cancer patients to ICI-targeted immunotherapy. One of these reported that an early elevation in CRP and/or systemic IL-6 within 7 days following administration of PD-1/PD-L1 inhibitors (nivolumab/pembrolizumab) to 31 evaluable patients with NSCLC “was indicated to be predictive of therapeutic efficacy” (64). This contention was based on the finding of ORRs of 0% (*n* = 0/7 patients) and 46% (11/24 patients) in patients with low and increased levels of CRP and/or IL-6, respectively (64). The corresponding median values for PFS were 112 days vs. not reached (*P* = 0.069). The frequencies of “severe (≥grade 3) adverse events” were 0% and 46% in patients with low and high levels of IL-6, respectively (64). Limitations of this study include its retrospective design, small number of patients and infrequent measurement of the test biomarkers.

Two other retrospective studies involving larger patient numbers have reported on the utility of measurement of CRP alone (65) and in combination with IL-6 (66) prior to initiation of ICI-based immunotherapy on the responses of cancer patients to ICI-targeted MAbs. In the first of these, Oya et al. reported that elevations in serum CRP of ≥10 micrograms/milliliter (μg/mL), recorded in 60 /124 patients with advanced NSCLC prior to administration of nivolumab, were associated with significant reductions in median PFS (1.8 vs. 4.0 months, *P* < 0.01, for patients with high and low CRP values, respectively) and OS (7.8 months vs. not reached, *P* < 0.01). However, associations with IRAEs were not recorded (65). More recently, Weber et al. analyzed both baseline and on-treatment levels of CRP and IL-6 in stored serum patients taken from melanoma patients who had participated in three different clinical trials (66). These patients had been treated with either ipilimumab or nivolumab individually, or in combination, or administered sequentially (nivolumab followed by ipilimumab). The authors observed that levels of CRP and IL-6 above the median values for these biomarkers at baseline were significantly associated with a poor response and decreased survival following therapy with nivolumab alone (66). In the case of ipilimumab alone, as well as combination therapy, similar findings were evident with respect to elevated CRP at baseline (66). Interestingly, *in vitro* mechanistic studies revealed that purified CRP, at concentrations of >10 micrograms/mL, caused significant suppression of T cell activation and proliferation (66). The authors concluded that “CRP and IL-6 are prognostic factors for checkpoint inhibition” (66). Although associations with IRAEs were not recorded in this summarized version of this study, they are likely to be included in the subsequent full publication of this congress abstract (66).

Clearly, additional studies, preferably prospective, are required to confirm the utility of the aforementioned, as well as other systemic biomarkers, in predicting responsiveness to ICI-targeted immunotherapy, as well as predisposition for development of IRAEs.

The following sections of this review are focused on possible mechanisms driving immune restoration following initiation of ICI-targeted immunotherapy, specifically the involvement of the gut microbiome and its role in promoting the differentiation of näive CD4+ T cells into mature pro-inflammatory Th17 cells.

## The Composition of the Gut Microbiome as a Determinant of the Immunorestorative Activity of CTLA-4- and PD-1-targeted Immunotherapy

Collectively, the extensive secondary lymphoid tissue lining the GIT has been estimated to account for up to 70% of the entire human immune system, making the gut mucosal immune system the largest lymphoid organ in the body, with most of the lymphocytes domiciled in the lamina propria (67, 68). In the GIT, the abundant cells of the adaptive, as well as innate, immune systems co-exist in apparent harmony with an estimated average 3.8 × 10^13^ commensal bacteria, located almost exclusively in the colon (69). Sustained immune homeostasis within the GIT therefore necessitates the maintenance of an environment of subdued immune reactivity necessary to protect and preserve beneficial microbial colonists. In this context, immunological tolerance is mediated in large part by the various subsets of CTLA-4-/PD-1-expressing Tregs. These cells abound in the intestinal mucosa and mesenteric lymph nodes, where their regulatory activities are augmented by other cell types such as anti-inflammatory subsets of dendritic cells, macrophages and innate lymphoid cells (70–74). Achieving a balanced environment is, however, critical to ensure prevention of excessive immunosuppression.

It is now well-accepted that in addition to their key roles in nutrient metabolism and absorption, that intestinal commensal microorganisms are critically involved in programming intestinal CD4+ and CD8+ T cells to effectively perform their protective functions, including their roles in anti-tumor immunity, an issue which appears to impact on the efficacy of ICI-targeted immunotherapy. This contention is supported by an accumulating body of evidence derived from clinical studies of cancer immunotherapy, especially those involving ICI-targeted MAbs, as well as from pre-clinical models of experimental tumorigenesis and infection. With respect to clinical studies highlighting the apparent involvement of the gut microbiota in promoting anti-tumor immunity during ICI-targeted immunotherapy, the study by Chaput et al. published in 2017, was among the first to describe this association (75). These researchers investigated the role of the baseline composition of the gut microbiome of patients with metastatic melanoma (*n* = 26) in predicting both clinical responsiveness and development of colitis during treatment with ipilimumab (75). They observed that patients colonized with the genus *Faecalibacterium* (phylum: *Firmicutes*, family: *Ruminococcaceae*) and other *Firmicutes* had significantly longer PFS (*P* = 0.0039) and OS (*P* = 0.051), as well as increased levels of systemic biomarkers of immune reactivity, in comparison with poor responders who were colonized predominantly with strains of the genus *Bacteroides* (75). As alluded to in the preceding section of this review, favorable clinical responses to ipilimumab in this study were also associated with an increased frequency of development of IRAEs, in this case, colitis (75).

The emerging strength of the association between the composition of the gut microbiota and the clinical responsiveness of patients with various types of advanced cancer to innovative immunotherapies, particularly those targeting ICIs, has been highlighted in several recent reviews (76–81). One of these (80), reviewed three major studies published simultaneously in “Science” in early January 2018 (82–84). These studies were focused on the association between different genera and species of colonic, commensal bacteria and the responsiveness of patients, mostly with metastatic melanoma or urothelial carcinoma, to PD-1/PD-L1-targeted immunotherapy (82–84).

Basically, the design of all three studies was somewhat similar, involving metagenomic analysis of the gut microbiome in relation to immune profiling of systemic and intra-tumoral indices of anti-tumor immunity, as well as clinical response (PFS) (82–84). All three studies noted significant associations between specific types of commensal organisms, anti-tumor reactivity, and clinical response (82–84). Overall, fourteen different bacterial genera and species were associated with favorable responses, although some inconsistencies were evident between studies (80). The most abundant colonic commensals in responders to PD-1-targeted immunotherapy detected in these studies were the genus *Faecalibacterium* (*Ruminococcaceae* family) (82); the species *Bifidobacterium longum, Colinsella aerofaciens*, and *Enterococcus faecium* (83), as well as *Akkermansia muciniphila* (84), all obligate anaerobes, with the exception of *E. faecium*, which is a facultative anaerobe. All three teams of investigators also demonstrated that fecal transplantation of germ-free mice with feces from human responders (cancer patients) who participated in the aforementioned studies, but not from non-responders, significantly enhanced the efficacy of PD-1-directed immunotherapy in murine models of experimental tumorigenesis (82–84).

In another very recent experimental animal study reported by Tanoue et al. the authors demonstrated that intestinal colonization of germ-free mice with eleven different species of essentially avirulent colonic bacteria, belonging predominantly to the order *Bacteroidales*, elicited the “robust” induction of IFN-γ-producing CD8+ cytotoxic T cells (85). This effect was dependent on the participation of colonic CD103^+^/MHC class Ia-expressing DCs, as well as significant increases in the numbers of colonic CD4 Th1 and Th17 cells (85). In the case of the induced intestinal CD8+ T cells, these cells migrated to distant anatomical sites, such as the spleen and liver, where they elicited protective immune responses against experimental infection with the bacterial pathogen, *Listeria monocytogenes* (85).

In the same study, these authors also investigated the potential of experimental colonization of germ-free mice with the same eleven strains of colonic commensal bacteria to potentiate PD-1 MAb-mediated anti-tumor immune responses in models of experimental skin tumorigenesis (adenocarcinoma and melanoma) (85). The authors observed that anti-tumor efficacy was associated with increased numbers of interferon (IFN)-γ-secreting CD8+ TILs and “was markedly more efficacious” in this respect (increased numbers of TILs) in mice treated with the combination of therapies, relative to the responses of those animals that received either the bacterial preparation or PD-1 MAbs individually (85). This type of combination immunotherapy was not accompanied by ICI-associated colitis, possibly due to a lower frequency of development of this IRAE following administration of PD-1 antagonists as opposed to that observed with MAbs that target CTLA-4 (85). The authors of this study conceded, however, that much additional work is necessary to characterize mechanisms of immunomodulation elicited by their strains of colonic, commensal bacteria, as well as realization of their biotherapeutic potential in combatting cancer in particular, as well as infectious diseases (85).

Adding to these findings is another very recent study by Li et al. who reported, that mice lacking the *Rnf5* gene, which encodes the RNF5 protein, ubiquitin ligase 5, involved in the unfolded protein response (UPR), manifested a gut microbiome strikingly distinct from that of their wild-type counterparts (86). The microbiome of mice lacking the *Rnf5* gene was “enriched” with the commensal bacterium, *Bacteroides rodentium* and was associated with immune alterations which included increased intestinal DC recruitment and activation and expression of inflammasome components (86). These immune alterations were positively associated with responsiveness to experimentally-induced melanoma, characterized by: (i) increased numbers of TILs of both the CD4 and CD8 phenotypes; (ii) increased reactivity of these cells, which was associated with enhanced production of IL-2, IFN-γ and tumor necrosis factor (TNF)-γ; and (iii) increased expression of MHC class II molecules, as well as the co-stimulatory molecules, B7.1 and B7.2, by DCs and tumor infiltrating macrophages (TIMs) (86). In agreement with the findings of the aforementioned study by Tanoue et al. (85), experimental intestinal colonization of germ-free mice with 11 bacterial strains predominant in the gut microbiome of mice lacking the *Rnf5* gene, including *B. rodentium*, was found to establish anti-tumor immunity according to restriction of growth of an experimental tumor (melanoma) (86).

In an extension of the aforementioned study, Li et al. also analyzed the mRNA expression levels of biomarkers of the UPR, specifically activating transcription factor 4 (ATF4), secreted immunoglobulin heavy chain-bindng protein1 (sBiP), and secreted X-box-binding protein 1 (sXBP1), in tumor biopsies from melanoma patients in relation to ICI-targeted immunotherapy (86). These experiments revealed that pre-treatment expression levels of all three of these UPR-related proteins were significantly (*P* < 0.05–*P* <0.005) lower in responders to CTLA-4-targeted immunotherapy relative to non-responders (a total of 23 and 32 patients, respectively, attending two different clinics) (86). The PFS rates were found to be significantly longer in patients with low expression levels of mRNA encoding sXBP1 and ATF4 (*P* < 0.0211 and *P* < 0.0076, respectively). In melanoma patients who qualified for PD-1-targeted therapy, low pre-treatment expression levels of BiP were significantly associated with prolonged OS (*P* = 0.008, *n* = 12 patients) and disease-free survival (*P* < 0.021, *n* = 9) (86).

The association between the composition of the gut microbiota and the anti-tumor efficacy of ICI-targeted immunotherapy is further strengthened by the findings of several studies, albeit with one exception (87), that prior or concomitant administration of antibiotics impacts negatively on the therapeutic benefit of CTLA-4- and PD-1/PD-L1-targeted MAbs (84, 88–90). In this context, co-administration for a period of 14 days of a cocktail of antibiotics (ampicillin + colistin + streptomycin) to mice housed in pathogen-free conditions and harboring experimentally-induced tumors (sarcoma/melanoma), resulted in significant attenuation of the anti-tumor efficacy of PD-1-MAbs administered alone or in combination with anti-CTLA-4 (84).

The same authors also investigated the effects of recent administration of antibiotics (β-lactams, fluoroquinolones, macrolides) on the anti-tumor efficacy of ICIs in two different clinical studies (84, 88). In the first of these, encompassing patients (*n* = 249) with advanced NSCLC (n = 42), renal cell carcinoma (RCC, *n* = 67) or urothelial carcinoma (*n* = 42), administration of antibiotics (*n* = 67 patients), either 2 months prior to, or 1 month after, initiation of PD-1/PD-L1-targeted ICI MAbs to the combined group of patients resulted in significant attenuation of immunotherapy-associated prolongation of PFS (antibiotic treated vs. untreated, 3.5 vs. 4.1 months, *P* = 0.017) and OS (11.5 vs. 20.6 months) (84). The second study, also encompassed patients (*n* = 360) with advanced NSCLC (*n* = 239) or RCC (*n* = 121), 48 and 16 of whom were treated with antibiotics within 30 days of commencement of immunotherapy, respectively. Patients with NSCLC were treated with PD-L1-based immunotherapy alone or in combination with anti-CTLA-4-targeted therapy, while PD1/PD-L1 MAbs were administered alone, or in combination with antI-CTLA-4-targeted therapy or the anti-angiogenic agent, bevacizumab, to those with RCC. In the combined group of patients, administration of antibiotics resulted in significant reductions in both PFS and OS relative to those patients who did not receive antibiotics (88). In the case of patients with NSCLC, the median PFS and OS values (antibiotic treated vs. untreated) were 1.9 vs. 3.8 months (*P* < 0.01) and 7.9 vs. 24.6 months (*P* < 0.01), respectively. The corresponding values for patients with RCC were 1.9 vs. 7.4 months (*P* < 0.01) and 17.3 vs. 30.6 months (88).

Similar findings have been reported in two other recent studies. The first of these was undertaken in patients with advanced NSCLC (*n* = 109) who received antibiotics (*n* = 20) either 1 month before, or within 1 month, of initiation of anti-PD-1-based therapy (89). Again, administration of antibiotics resulted in significant shortening of both PFS and OS, with respective median values (antibiotic treated vs. untreated) of 3.73 vs. 9.63 months, *P* < 0.0001 and 6.07 vs. 21.87 months, *P* = 0.004 (89). The second study involved patients with advanced melanoma (*n* = 74), ten of whom received antibiotics within 30 days of administration of anti-PD-1- and/or anti-CTLA-4 MAb-targeted therapies (90). In this study, the respective median PFS and OS values (antibiotic treated vs. untreated) were 2.4 vs. 7.3 months (*P* = 0.01) and 10.7 vs. 18.3 months (*P* = 0.17) (90).

Although the findings of the aforementioned studies are clearly consistent with an association between the composition of the gut microbiome and responsiveness to ICI MAb-based anti-cancer therapy and possibly development of IRAEs, they should, however, be confirmed in appropriately designed prospective clinical studies. In our opinion, studies of this nature should be undertaken together with companion translational studies focused on identifying the mechanisms underpinning these associations.

## Potential Mechanisms Underpinning Immune Restoration and the Development of IRAEs During Immune Checkpoint Inhibitor-Targeted Therapy

Although T cell-mediated immune mechanisms appear to predominate, particularly decreased Tregs and other types of immunosuppressive cells, the immunopathogenesis of a number of IRAEs is likely to be multifactorial, a contention which is based on the range of autoinflammatory/autoimmune disorders precipitated or exacerbated by ICI-based immunotherapy. This may be most evident in the case of PD-1-targeted therapy, given the broader level of expression this ICI relative to that of CTLA-4, which encompasses various cell types of both the adaptive and innate immune systems. In the case of the former, these include B cell/autoantibody/complement-driven mechanisms, which may underpin development or exacerbation of existing of autoimmune disorders such as myasthenia gravis (91–93), autoimmune hemolytic anemia (94–97), and type 1 diabetes mellitus (98). With respect to mechanisms involving cells of the innate immune system such as DCs, monocytes/macrophages and granulocytes, as well as structural cells, including fibroblasts and epithelial cells, activation of the NLRP3 (NOD-like receptor family pyrin domain containing 3) inflammasome may contribute to the pathogenesis of IRAEs. In this setting, activation of the NLRP3 inflammasome may occur as a consequence of release of alarmins/danger-associated molecular patterns (DAMPS), such as high-mobility group box 1 protein (HMGB1), from dead and dying tissue cells (99). In addition, perforins released by activated cytotoxic T cells and natural killer (NK) cells have been reported to activate the NLRP3 inflammasome in DCs and presumably other cell types (100). These mechanisms involving the NLRP3 inflammasome may also impact on the type and severity of IRAEs.

Notwithstanding the types of mechanism underpinning immunpathogenesis, the preceding sections of this review have highlighted two emerging aspects of ICI MAb-mediated anti-cancer therapy and development of IRAEs. These are firstly, that the beneficial anti-tumor activity and associated development of IRAEs appear to share a common origin, consistent with an overall, as opposed to selective, enhancement of immune reactivity (101). This contention is supported by findings that administration of ipilimumab or PD-1-targeted MAbs to patients with advanced melanoma is accompanied by rapid diversification of the T cell repertoire (102–104), described by the authors of one of these studies as being “both detrimental and beneficial for patients with cancer” (102). Secondly, following ICI MAb-mediated targeting of CTLA4-/PD-1-expressing Tregs, effective recovery of anti-tumor immunity, and possibly development of IRAEs, appears to be dependent on a gut microbiota populated with commensal microorganisms conducive to immune system re-programming (75–86, 88–90).

Although the intricacies of immune system recovery following administration of CTLA-4- and PD-1-targeted MAbs, individually or in combination, remain to be fully elucidated, it seems likely that the differentiation and expansion of gut mucosal Th17 cells, which are strongly influenced by components of the gut microbiota (24), play a prominent role in the pathogenesis of some types of IRAEs and possibly the anti-tumor effects of these agents. This contention, which represents the focus of the remaining sections of this review, is based on several lines of evidence as follows:

- Th17 cells originating in the gut migrate to distant anatomical sites where they have been implicated in the pathogenesis of various autoimmune and auto-inflammatory disorders in both the experimental and clinical settings, including colitis, multiple sclerosis, and rheumatoid arthritis (24, 105–107). This may be exacerbated by the phenomenon of T cell plasticity, which, depending on the DC and cytokine environment, enables Th17 cells to acquire a dual Th1/Th17 phenotype, resulting in co-expression of IL-17, and IFN-γ (108);
- patients with metastatic melanoma treated with tremelimumab (CTLA-4-targeted MAb), either alone or in combination with other types of immunotherapy, demonstrated increased numbers of circulating Th17 cells, measured as IL-17A-secreting CD4+ T cells following activation *in vitro* (109). Expansion of these cells was associated with an increased frequency of inflammatory and autoimmune toxicities (colitis, hypophysitis) (109);
- the intestinal lamina propria of germ-free mice is populated extensively by Tregs, with few Th17 cells, which are normally “selectively and constitutively” present in the lamina propria of wild-type animals (110, 111);
- introduction into the gut lumen of germ-free mice of a cocktail of pathogen-free, gut commensal bacteria, containing members of the *Cytophaga-Flavobacter-Bacteroides* phylum, was found to induce the differentiation of Th17 cells in the mucosa of the small intestine (111); similar effects were observed following experimental intestinal colonization of germ-free mice with symbiont bacterial species isolated from human gut, particularly the species, *Bifidobacterium adolescentis* (112);
- rectal or systemic administration of adenosine 5′-triphosphate (ATP), which is produced in high concentrations by gut commensal bacteria, but is present in relatively low concentrations in the intestinal lumen of germ-free mice, drives the differentiation of lamina propria Th17 cells, which is associated with exacerbation of T cell-mediated colitis (110). Mechanisms by which intestinal, microbial-derived ATP modulates gut immune homeostasis and differentiation of Th17 cells are explored further in the following section.

## Role of ATP Derived From Resident Commensal Bacteria in Promoting Differentiation of Intestinal Th17 Cells

As mentioned above, the intestinal lumen contains high levels of ATP released from trillions of gut-colonizing, commensal bacteria, estimated to encompass around 1,000 different species (113). Microbial-derived ATP, in turn, appears to play a key role in creating a cytokine environment conducive to promoting the differentiation of naïve CD4 T cells into mature, pro-inflammatory Th17 cells (110, 113). This transition is dependent on the interaction of ATP with a unique subset of lamina propria DCs of the phenotype CD70 (high), CD11c (low) (85), which are also known as DC17 cells (114). The profile of cytokines secreted by these cells, namely IL-1β, IL-6, IL-23, together with the expression of the α5/β8 integrin, which promotes activation of latent pro-TGF-β1, drives the differentiation of näive CD4 T cells into Th17 cells (110, 114). ATP-mediated activation of Th17 cell-inducing DC cell subsets, has also been reported to occur in the skin (114) and visceral adipose tissue (115), driving psoriasis and obesity-induced inflammation, respectively.

The mechanisms involved in ATP-mediated secretion of pro-inflammatory cytokines appear to involve interaction of this purine nucleotide with the purinergic P2X7 receptor. This receptor is an extracellular, ATP-gated channel expressed on immature DC subsets, which promotes the migration of these cells, as well as their maturation into the DC17 phenotype, driving Th17 cell polarization (110, 114–116). As mentioned above, this is achieved via the synthesis of pro-inflammatory cytokines (115), as well as by activation of the NLRP3 inflammasome, resulting in generation of functional IL-1β (116).

In the quiescent colon, ATP-driven differentiation of Th17 cells may, however, be counteracted by intestinal Tregs. In addition to the various immunosuppressive activities of Tregs mentioned earlier, these cells have also been implicated in regulating ATP-driven immune and inflammatory responses. In this context, subsets of both murine and human Foxp3^+^ Tregs have been reported to express CD39, an ATP hydrolyzing enzyme (nucleoside triphosphate diphosphohydrolase-1), which depletes ATP via conversion to adenosine monophosphate (AMP) (20–22). In addition to depletion of ATP, augmentation of the immunosuppressive activity of CD39-expressing Tregs is achieved via co-expression of a second nucleotidase *viz*. CD73 (ecto-5′-nucleotidase), which converts AMP to immunosuppressive adenosine (20–22). In this setting, adenosine, via interaction with adenylyl cyclase-coupled A2_A_ receptors expressed on cells of both the innate and adaptive immune systems, triggers intracellular synthesis of broadly immunosuppressive adenosine 3′,5′-cyclic monophosphate (cyclic AMP).

Although plausible, the relevance of these Treg-mediated, ATP-targeted mechanisms in maintaining gut immune homeostasis in humans remains to be established, as does the possible role of CTLA-4/PD-1-targeted immune checkpoint inhibition in negating these mechanisms of immunosuppression.

Clearly, other bacterial-derived pro-inflammatory agents such as nucleic acids and cell-wall components, are also likely to accrue following attenuation of Treg-mediated immunosuppression during ICI-targeted immunotherapy. These are also potential contributors to the activation of intestinal DCs via interaction with pattern recognition receptors (PRRs), driving differentiation and activation of Th17 cells.

**Figure 1:**
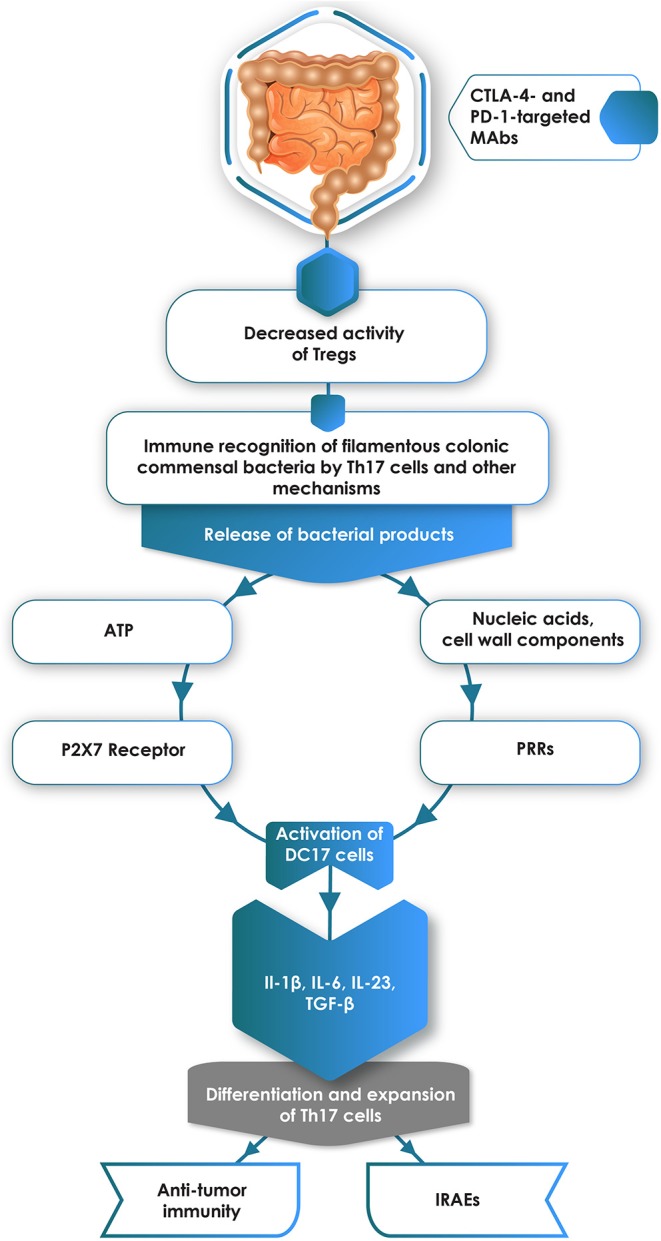
Administration of CTLA-4- and/or PD-1-targeted monoclonal antibodies (MAbs) attenuates the immunosuppressive activity of intestinal Tregs. Relief from Treg-mediated immunosuppression enables immune recognition of colonic commensal bacteria, particularly filamentous anaerobes, with resultant release of pro-inflammatory bacterial components, particularly ATP and nucleic acids/cell wall components, which interact with P2X7 receptors and pattern recognition receptors (PRRs), respectively expressed on immature DC17 cells. This, in turn, results in activation of these cells, creating a cytokine environment conducive to the differentiation of naïve CD4^+^ T cells into pro-inflammatory Th17 cells. These cells are implicated in the pathogenesis of some types of immune-related adverse events (IRAEs), but have divergent effects on anti-tumor immunity.

On the basis of the evidence presented in this section of the review, a possible scenario whereby ICIs drive the differentiation of intestinal Th17 cells with resultant susceptibility for development of some types of IRAEs is shown in Figure 1.

## Th17 Cells and Anti-tumor Immunity

Notwithstanding the probable involvement of Th17 cells in the immunopathogenesis of some types of IRAEs, the role of these cells in promoting effective anti-tumor immunity does, however, remain somewhat contentious. In this context, earlier studies based on murine models of experimental tumorigenesis (cutaneous and lung melanoma) appeared to be supportive of a protective role for Th17 cells in anti-tumor host defenses (117, 118). Although not appearing to possess direct anti-tumor activity, Th17 cells in the tumor microenvironment were found to promote the influx of monocytes/macrophages, DCs, NK cells and effector, memory CD4+ and CD8+ T cells via secretion of the chemokines CCL2 and CCL20 (117, 118). In addition, to recruiting CD8+ cytotoxic T cells, Th17 cells also promoted activation and expansion of these cells (117, 118). Other potential mechanisms of Th17 cell-mediated anti-tumor immunity include T cell plasticity with resultant acquisition by Th17 cells of the dual Th1/Th17 phenotype and production of cytotoxic T cell-activating IFN-γ (108).

It is now apparent, however, that Th17 cells play divergent roles in the immunpathogenesis of cancer, promoting both anti-tumor immunity and tumorigenesis (119, 120). With respect to pro-tumorigenic activity, IL-17A, which is produced not only by Th17 cells, but also by tumor cells *per se* (119), has been identified as a major offender. In this context, IL-17A has been found to promote tumor cell growth and proliferation, angiogenesis via production of vascular endothelial growth factor (VEGF) by tumor cells, as well as invasion and metastasis via production of matrix metalloproteinase-9 by tumor cells and other cell types (119, 121, 122). The potential threat to the clinical efficacy of immunotherapy posed by generation of excessive levels of IL-17A in the tumor microenvironment, both preceding and during anti-cancer chemotherapy, has been demonstrated in the clinical settings of the estrogen receptor-negative and triple-negative/basal-like subtypes of breast cancer (123, 124), thyroid cancer (125) and colorectal cancer (126). In the case of the latter type of malignancy, co-administration of anti-IL-17A/IL-17AR MAbs has been proposed as a potential adjuvant strategy to sustain the efficacy of MAb-based anti-VEGF therapy in metastatic disease (127).

Additional evidence implicating the involvement of IL-17A in tumorigenesis has also been derived from murine models of experimental colon cancer. In one study, deletion of the genes encoding either IL-17A or its receptor (IL-17AR) was found to confer protection against tumor promotion and progression, while combining administration of an anti-IL-17A MAb together with chemotherapy was found to enhance therapeutic efficacy (128). Similar findings have also been reported in genetically engineered mice harboring KRAS (Kirsten rat sarcoma viral oncogene homolog) mutation–positive NSCLC co-expressing IL-17A, which resulted in augmentation of neutrophil infiltration and tumor progression, as well as decreased efficacy of PD-1-targeted immunotherapy (129).

To date, however, we are not aware of completed clinical trials which have focused on the administration of MAbs targeting either IL17A or its receptor as a potential strategy to enhance the efficacy of anti-tumor chemotherapy and/or ICI-based immunotherapy.

## Immune-based Strategies to Optimize Immune Checkpoint Inhibitor-based Therapy

According to the findings of their recently published survey based on cancer patients treated with ICI-targeted MAbs in the US during the period 25 March 2011–17 August 2018, Haslam and Prasad reported that six of these agents were approved for treatment of fourteen different malignancies (see Supplementary Table for registered indications for ICI-based cancer therapy) (130). The authors estimated that the percentage of cancer patients responsive to ICI-targeted MAbs was 0.14% in 2011 when ipilimumab was approved for treatment of unresectable or metastatic melanoma, increasing to 5.86% by 2015. The overall percentage of responders was estimated to reach 12.46% in 2018 (130), which is lower than that reported in studies cited in earlier sections of this review. The “indications that contributed most to the response estimate included NSCLC (7.09%), renal cell carcinoma (1.02%), and melanoma (0.92%).” Response to therapy was “based on the best available response rate (FDA drug label) for that indication,” which may account in part for the lower than expected response rate (130). Although the response rates were relatively low, most responders attained durable responses and had significant clinical benefit. Haslam & Prasad concluded that the estimated percentages of patients potentially eligible for ICI treatment and their responses to this type of therapy, are superior to the reported estimates for agents approved for genome-driven oncotherapy. However, at an estimated cost of 7 billion dollars, the percentage of patients who benefit from these treatments remains modest (130) and therefore future research should investigate the use of predictive biomarkers to maximize the benefit of immunotherapy among cancer patients undergoing these treatments (130).

Notwithstanding identification of reliable biomarkers predictive of responses to ICI-based cancer therapy, these findings again highlight the importance of identifying strategies to improve the efficacy of ICI MAb-based anti-tumor immunotherapy in the setting of minimizing the occurrence of IRAEs. Despite the considerable magnitude of this challenge, the aforementioned emerging insights into both the mechanisms and obstacles underpinning successful ICI MAb-mediated immunorestoration, have identified potential strategies to achieve this goal. These are distinct from immunosuppressive strategies currently used to control IRAEs, including administration of corticosteroids or MAbs such as infliximab or vedolizumab, and are summarized as follows (131).

### Combination Therapy

This strategy involves combining ICI MAb-based immunotherapy with agents that induce immunogenic cell death, such as certain chemotherapeutic agents and or radiation therapy, to intensify localized anti-tumor immune responses. Many clinical trials addressing these strategies are currently in progress (132, 133). The results of one such trial, the TONIC trial, have been published recently (133). This was an adaptive, non-comparative phase 2 trial involving patients (*n* = 66) with metastatic triple-negative breast cancer, a type of malignancy which is poorly responsive to PD-1/PD-L1-targeted immunotherapy. Patients were randomized to receive either no induction (*n* = 12), or induction for 2 weeks with low-doses of various inducers of immunogenic cell death *viz.*, irradiation of a single lesion (*n* = 12), cisplatin (*n* = 13), cyclophosphamide (*n* = 12), or doxorubicin (*n* = 17), followed by treatment with nivolumab (134). The authors observed that the most favorable overall response rates to nivolumab were evident in those patients who had been induced with low-dose doxorubicin in particular, as well as cisplatin, and were associated with transition to a more immunoreactive tumor microenvironment. While conceding various limitations associated with their study, particularly the necessity for stringent validation, the authors contend that their findings provide support for combination immunotherapy not only in breast cancer, but also in the broader context (134). This contention is supported by the findings of a recent randomized, stage 3 prospective study, which showed that the combination of atezolizumab plus nab-paclitaxel was associated with a prolongation of PFS “among patients with metastatic triple-negative breast cancer in both the intention-to-treat population and the PD-L1–positive subgroup” (135).

With respect to development of IRAEs in the setting of ICI-based immunotherapy together with chemotherapeutic agents, this type of combination therapy does not appear to be associated with an increased incidence of IRAEs. This contention is based on the findings of the KEYNOTE-189 clinical trial, a phase III clinical study which enrolled 616 patients with advanced non-squamous NSCLC and randomized patients in a 2:1 ratio to receive chemotherapy, consisting of cisplatin or carboplatin with pemetrexed with or without pembrolizumab (136). At a median follow-up of 10.5 months, the addition of pembrolizumab to chemotherapy resulted in improved OS rates relative to chemotherapy alone (69 vs. 49 percent). Grade 3 IRAEs or higher occurred in 67.2 and 65.8% of patients in the pembrolizumab-combination and the placebo-combination groups, respectively. In this large study phase III study, the combination of ICI and platinum-based chemotherapy was not associated with an unexpectedly high incidence of IRAEs (136).

### Manipulation of the Gut Microbiome

Tanoue et al. as described above (85), albeit using murine models of experimental skin tumorigenesis in germ-free mice, reported that intestinal colonization with a cocktail of 11 different avirulent commensal bacteria caused significant augmentation of PD-1 MAb-mediated anti-tumor activity (85). Importantly, this was associated with infiltration and expansion of IFN-γ-producing CD8+ cytotoxic TILs in the absence of colitis (85). However, given the potential risk of infection with multidrug-resistant pathogens associated with fecal transplantation, this strategy clearly requires caution and refinement.

### Pharmacological Alteration of the Phenotype and Functionality of Th17 Cells

This strategy, which admittedly is based on promising data derived from preclinical studies, is based on a study by Majchrzak et al. (137). These authors reported that murine ICOS (inducible T cell co-stimulator)-induced Th17 cells transitioned to a low IL-17A-secreting phenotype following treatment with inhibitors of phosphatidylinostitol 3-kinase δ (idelalisib) and β-catenin (indomethacin) (137). Adoptive transfer of these cells resulted in augmentation of anti-tumor activity in a murine model of experimental tumorigenesis (137).

### Potential Utility of IL-17A-Targeted Monoclonal Antibodies

Given the apparent association of Th17 cells and IL-17A with some types of IRAEs, co-administration of IL-17A/IL17AR MAbs together with ICI-targeted MAbs may not only enhance anti-tumor immunity, but also attenuate or prevent the development of IRAEs. In this context, MAbs targeting IL-17A (ixekizumab, secukinumab) or its receptor (brodalumab) are currently used in the immunotherapy of various types of autoinflammatory/autoimmune diseases, including ankylosing spondylitis, inflammatory bowel disease, multiple sclerosis, psoriasis, psoriatic arthritis, and rheumatoid arthritis (138). To our knowledge, however, and as mentioned above, there are no published clinical trials focused on the possible utility of IL-17A/IL-17AR-targeted MAbs in either the immunotherapy of human cancers, or as a strategy to counter development of IRAEs during ICI-based therapy. There are, however, two case reports which have documented the utility of administration of the IL-17A-targeted MAb, secukinumab, in causing resolution of pembrolizumab-associated, severe psoriasiform dermatologic toxicity in one patient with metastatic colon cancer (139) and a second with metastatic melanoma (140). Clearly, additional studies are necessary to confirm these findings in larger cohorts of cancer patients receiving different types of ICI-targeted MAbs associated with a broader spectrum of IRAEs. Attention to safety issues, particularly increased susceptibility for development of microbial infection, is also a priority.

### Potential Utility of IL-1β-Targeted Monoclonal Antibodies

A potential alternative strategy to that based on IL-17A-targeted monoclonal antibodies involves targeting cytokines that drive Th17 maturation/differentiation, specifically IL-1β, IL-6, and IL-23. In this context, it is noteworthy that administration of the IL-1β-targeted MAb, canakinumab, to patients (*n* = 10,061) with atherosclerosis, who had experienced a prior myocardial infarction and had no previously diagnosed cancer, was associated with significant (*P* = 0.034–*P* < 0.0001), dose-related reductions in the subsequent incidence of lung cancer (141, 142). These findings have led to initiation of the CANOPY program encompassing three phase III clinical trials (143). These are: (i) the CANOPY-A trial (“Study of Efficacy and Safety of Canakinumab as Adjuvant Therapy in Adult Subjects with Stages AJCC/UICC v. II-IIIA (T>5 cm N2) Completely Resected Non-small Cell Lung Cancer”; NCT03447769) (144); (ii) CANOPY−1 (“Study of Efficacy and Safety of Pembrolizumab Plus Platinum-based Doublet Chemotherapy With or Without Canakinumab in Previously Untreated Locally Advanced or Metastatic Non-squamous Squamous NSCLC Subjects”; NCT03631199) (145); and (iii) CANOPY-2 (“Phase III Study Evaluating Efficacy and Safety of Canakinumab in Combination With Docetaxel in Adult Subjects With Non-small Cell Lung Cancers as a Second or Third Line Therapy”; NCT03626545 (146). Although primarily focused on the therapeutic efficacy of canakinumab with and without different types of chemotherapy in patients with NSCLC, the outcome of the CANOPY Program clinical trials, specifically CANOPY-1, will also be of interest with respect to the incidence of IRAEs. In this context, it is noteworthy that targeting of pro-inflammatory cytokines, including, not only IL-1β and IL-17A, but also IL-6, IL-12, IL-23 and TNF-α, as a strategy in the management of different types of refractory ICI-related toxicities, has also recently been advocated by Martins et al. (147).

Irrespective of the therapeutic potential of the aforementioned strategies aimed at optimizing ICI-based immunotherapy, much additional research in both the experimental and clinical settings needs to be undertaken.

## Conclusions

Increasing awareness of the apparent overactivity of the immune system during administration of ICI-targeted MAbs as the cause of both the beneficial and harmful effects of this type of immunotherapy has underscored not only the importance, but also the difficulty, of formulating potential strategies to dissociate these activities. Although corticosteroids and other immunosuppressive agents are useful in the management of IRAEs, this must be counterbalanced against the risk of opportunistic infections, including tuberculosis, as well as attenuation of the beneficial immunorestorative effects of ICI-targeted therapy. Alternative strategies which may subdue IRAEs in the setting of retention of anti-tumor activity are, however, mostly in the pre-clinical stages of evaluation. A notable exception, currently in the advanced stages of clinical evaluation, is the strategy of combining ICI-targeted MAbs with inducers of immunogenic cell death (chemotherapeutic agents and/or radiotherapy), which may intensify localized anti-tumor responses. Promising strategies currently in the pre-clinical or early clinical phases of evaluation include administration of gut microbiota-based biopharmaceuticals and modulating the pro-inflammatory activities of Th17 cells via MAb-based targeting of IL-17A or its receptor.

## Author Contributions

All authors listed have made a substantial, direct and intellectual contribution to the work, and approved it for publication.

### Conflict of Interest

The authors declare that the research was conducted in the absence of any commercial or financial relationships that could be construed as a potential conflict of interest.
