# Supplementary material for: Immunopathogenesis of Immune Checkpoint Inhibitor-Related Adverse Events: Roles of the Intestinal Microbiome and Th17 Cells
Source: Front Immunol. 2019 Sep 26;10:2254. doi: 10.3389/fimmu.2019.02254 (PMC6775220; doi:10.3389/fimmu.2019.02254)
Supplement: Supplementary file 1 [file Table_1.docx]

**SUPPLEMENTARY TABLE WITH REFERENCES**

**Approved antibodies blocking Programmed death-1 (PD-1)/CTLA-4 and indications for use:**

**Pembrolizumab**

**Malignant Melanoma**

- **First line metastatic setting:** For the treatment of patients with unresectable or metastatic melanoma [1].
- **Adjuvant setting:** For adjuvant treatment of patients with melanoma with involvement of lymph node(s) or following complete resection [2].

**Non-Small Cell Lung Cancer (NSCLC)**

- **First line metastatic setting:** In combination with pemetrexed and platinum-based chemotherapy treatment of patients with metastatic non-squamous NSCLC and no epidermal growth factor receptor (EGFR) or anaplastic lymphoma kinase (ALK) genomic tumor mutations detected [3];
- **First line metastatic setting:** In combination with carboplatin and either paclitaxel or nab-paclitaxel for treatment of patients with metastatic squamous NSCLC [4-5];
- **First line metastatic setting:** As a single agent for the first-line treatment of patients with metastatic NSCLC with high PD-L1 expression [Tumor Proportion Score (TPS) ≥50%] as determined by an FDA-approved test, and with no EGFR or ALK genomic tumor mutations detected [6];
- **Second line metastatic setting:** As a single agent for the treatment of patients with metastatic NSCLC (squamous and non-squamous) whose tumors express PD-L1 (TPS ≥1%) as determined by an FDA-approved test, with disease progression on or following platinum-based chemotherapy. Patients with EGFR or ALK genomic tumor mutations should have disease progression on FDA-approved treatment for these mutations prior to receiving pembrolizumab [6].

**Head and Neck Squamous Cell Cancer (HNSCC)**

- **Second line metastatic setting:** For the treatment of patients with recurrent or metastatic HNSCC with disease progression on or following platinum-based chemotherapy treatment [7].

**Classical Hodgkin’s Lymphoma (cHL)**

- **Relapsed setting:** For the treatment of adult and pediatric patients with refractory cHL, or who have relapsed following 3 or more prior lines of treatment [8].

**Primary Mediastinal Large B-Cell Lymphoma (PMBCL)**

- **Relapsed setting:** For the treatment of adult and pediatric patients with refractory PMBCL who have relapsed following 2 or more prior lines of treatment [9].

**Urothelial Carcinoma**

- **First line metastatic setting:** For the treatment of patients with locally advanced or metastatic urothelial carcinoma who are not eligible for cisplatin-based chemotherapy and whose tumors express PD-L1 [Combined Positive Score (CPS) ≥10] as determined by an FDA-approved test, or in patients who are not eligible for any platinum-based chemotherapy regardless of PD-L1 status [10];
- **Second line metastatic setting:** For the treatment of patients with locally advanced or metastatic urothelial carcinoma who have disease progression during or following platinum-based chemotherapy [11];
- **First line metastatic setting:** For the treatment of patients who relapse within 12 months of neoadjuvant or adjuvant treatment with platinum-based chemotherapy [11].

**Microsatellite Instability-High Cancer**

- **Relapsed metastatic setting:** For the treatment of adult and pediatric patients with unresectable or metastatic, microsatellite instability-high (MSI-H) or mismatch repair deficient solid tumors that have progressive disease following prior treatment and who have no satisfactory alternative treatment options;
- **Relapsed metastatic setting:** For colorectal cancer that has progressive disease following treatment with a fluoropyrimidine, oxaliplatin, and irinotecan based chemptherapy treatment [12].

**Gastric Cancer**

- **Relapsed metastatic setting:** For the treatment of patients with recurrent, locally advanced or metastatic gastric or gastroesophageal junction adenocarcinoma whose tumors express PD-L1 CPS ≥1 as determined by an FDA-approved test, with progressive disease on or after two or more prior lines of treatment including fluoropyrimidine- and platinum-based chemotherapy and, if appropriate, anti-HER2/neu-targeted therapy [13-14].

**Cervical Cancer**

- **Second line metastatic setting:** For the treatment of patients with recurrent or metastatic cervical cancer with disease progression on, or after chemotherapy, whose tumors express CPS ≥1 as determined by an FDA- approved test [15].

**Hepatocellular Carcinoma**

- **Second line metastatic setting:** For the treatment of patients with hepatocellular carcinoma who have been previously treated with sorafenib [16].

**Merkel Cell Carcinoma**

- **Second line metastatic setting:** For the treatment of adult and pediatric patients with recurrent locally advanced or metastatic Merkel cell carcinoma [17].

**Nivolumab**

**Malignant Melanoma**

- **First line metastatic setting:** As a single agent or in combination with ipilimumab, is indicated for the treatment of patients with unresectable or metastatic melanoma [18-20].
- **Adjuvant setting:** For the adjuvant treatment of patients with melanoma with involvement of lymph nodes or metastatic disease who have undergone complete resection [21].

**Non-Small Cell Lung Cancer**

- **Second line metastatic setting:** For the treatment of patients with metastatic non-small cell lung cancer (NSCLC) with progression on or after platinum-based chemotherapy. Patients with EGFR or ALK genomic tumor mutations should have disease progression on FDA-approved treatment for these mutations prior to receiving nivolumab [22-24].

**Small Cell Lung Cancer**

- **Second line metastatic setting:** For the treatment of patients with metastatic small cell lung cancer (SCLC) with disease progression following platinum-based chemotherapy and at least one other line of treatment [25].

**Renal Cell Carcinoma**

- **Second line metastatic setting:** For the treatment of patients with advanced renal cell carcinoma (RCC) who have received prior anti-angiogenic treatment [26].
- **First line metastatic setting:** In combination with ipilimumab, is indicated for the treatment of patients with intermediate or poor risk, previously untreated RCC [27].

**Hodgkin’s Lymphoma**

- **Relapsed setting:** For the treatment of adult patients with cHL that has relapsed or progressed after autologous hematopoietic stem cell transplantation (HSCT) and brentuximab vedotin, or three or more lines of systemic treatment that include autologous HSCT [28-29].

**Squamous Cell Carcinoma of the Head and Neck**

- **Second line metastatic setting:** For the treatment of patients with recurrent or metastatic squamous cell carcinoma of the head and neck (SCCHN) with disease progression on or after platinum-based treatment [30].

**Urothelial Carcinoma**

- **Second line metastatic setting:** For the treatment of patients with locally advanced or metastatic urothelial carcinoma who have disease progression during or following platinum-based chemotherapy, have disease progression within 12 months following neoadjuvant or adjuvant treatment with platinum-based chemotherapy [31].

**Microsatellite Instability-High (MSI-H) or Mismatch Repair Deficient (dMMR) Metastatic Colorectal Cancer**

- **Relapsed setting:** As a single agent, is indicated for the treatment of adult and pediatric patients, 12 years and older with microsatellite instability-high (MSI-H) or mismatch repair-deficient (dMMR) metastatic colorectal cancer (CRC) that has progressive disease following treatment with a fluoropyrimidine, oxaliplatin, and irinotecan [32];
- **Relapsed setting:** In combination with ipilimumab, is indicated for the treatment of adults and pediatric patients (12 years and older) with MSI-H or dMMR metastatic CRC that has progressive disease following treatment with a fluoropyrimidine, oxaliplatin, and irinotecan [33].

**Hepatocellular Carcinoma**

- **Second line metastatic setting:** For the treatment of patients with hepatocellular carcinoma who have been previously treated with sorafenib (second line setting) [34].

**Cemiplimab**

**Cutaneous squamous cell carcinoma**

- **First line metastatic setting:** For the treatment of patients with metastatic cutaneous squamous cell carcinoma (CSCC) or locally advanced CSCC who are not candidates for curative surgery or curative radiation [35.]

**Antibodies blocking Programmed death-ligand 1 (PD-L1) and indications for use:**

**Atezolizumab**

**Urothelial Carcinoma**

- **First line metastatic setting:** For the treatment of patients with locally advanced or metastatic urothelial carcinoma who are not eligible for cisplatin-based chemotherapy, and whose tumors express PD-L1 (PD-L1 stained tumor-infiltrating immune cells [IC] covering ≥ 5% of the tumor area), or are not eligible for any platinum-based chemotherapy regardless of level of tumor PD-L1 expression;
- **Second line metastatic setting:** For patients who have disease progression during or following any platinum-based chemotherapy, or within 12 months of neoadjuvant or adjuvant chemotherapy [36].

**Non-Small Cell Lung Cancer**

- **Second line metastatic setting:** For the treatment of patients with metastatic NSCLC who have disease progression during or following platinum-based chemotherapy. Patients with EGFR or ALK genomic tumor mutations should have disease progression on FDA- approved treatment for these mutations prior to receiving atezolizumab [37].

**Durvalumab**

**Urothelial carcinoma**

- **Second line metastatic setting:** For the treatment of patients with locally advanced or metastatic urothelial carcinoma who have disease progression during or following platinum-based chemotherapy or have disease progression within 12 months of neoadjuvant or adjuvant treatment with platinum-based chemotherapy [38.]

**Non-Small Cell Lung Cancer**

- **Adjuvant setting:** Unresectable, Stage III NSCLC in which the disease has not progressed following concurrent platinum-based chemotherapy and radiation therapy [39].

**Avelumab**

**Merkel Cell Carcinoma**

- **First line metastatic setting:** For the treatment of adults and pediatric patients 12 years and older with metastatic Merkel cell carcinoma [40].

**Antibodies blocking cytotoxic T lymphocyte -4 (CTLA-4) and indications for use:**

**Ipilimumab**

**Malignant Melanoma**

- **First line metastatic setting:** For the treatment of unresectable or metastatic melanoma in adults and pediatric patients (12 years and older) [19];
- **Adjuvant setting:** For the adjuvant treatment of patients with cutaneous melanoma with pathologic involvement of regional lymph nodes of more than 1 mm who have undergone complete resection, including total lymphadenectomy [41].

**Renal Cell Carcinoma**

- **First line metastatic setting:** In combination with nivolumab, for the treatment of patients with intermediate or poor risk, previously untreated advanced renal cell carcinoma (RCC) [26].

1. Schachter J, Ribas A, Long GV, Arance A, Grob JJ, Mortier L, et al. Pembrolizumab versus ipilimumab for advanced melanoma: final overall survival results of a multicentre, randomised, open-label phase 3 study (KEYNOTE-006). *Lancet*. (2017) 390:1853–1862. doi: 10.1016/S0140-6736(17)31601-X.
2. Eggermont AMM, Blank CU, Mandala M, Long GV, Atkinson V, Dalle S, et al. Adjuvant pembrolizumab versus placebo in resected stage III melanoma. *N Engl J Med*. (2018) 378:1789–1801. doi: 10.1056/NEJMoa1802357.
3. Gandhi L, Rodríguez-Abreu D, Gadgeel S, Esteban E, Felip E, De Angelis F, et al; KEYNOTE-189 Investigators. Pembrolizumab plus chemotherapy in metastatic non-small-cell lung cancer. *N Engl J Med.* (2018) 378:2078–2092. doi: 10.1056/NEJMoa1801005.
4. Reck M, Rodríguez-Abreu D, Robinson AG, Hui R, Csőszi T, Fülöp A, et al; KEYNOTE-024 Investigators. Pembrolizumab versus chemotherapy for PD-L1-positive non-small-cell lung cancer. *N Engl J Med.* (2016) 375:1823–1833.
5. Paz-Ares L, Luft A, Vicente D, Tafreshi A, Gümüş M, Mazières J, et al; KEYNOTE-407 Investigators. Pembrolizumab plus chemotherapy for squamous non-small-cell lung cancer. *N Engl J Med*. (2018) 379:2040–2051. doi: 10.1056/NEJMoa1810865.
6. Pai-Scherf L, Blumenthal GM, Li H, Subramaniam S, Mishra-Kalyani PS, He K, et al. FDA Approval Summary: pembrolizumab for treatment of metastatic non-small cell lung cancer: first-line therapy and beyond. *Oncologist.* (2017) 22:1392–1399. doi: 10.1634/theoncologist.2017-0078.
7. Cohen EEW, Soulières D, Le Tourneau C, Dinis J, Licitra L, Ahn MJ, et al; KEYNOTE-040 investigators. Pembrolizumab versus methotrexate, docetaxel, or cetuximab for recurrent or metastatic head-and-neck squamous cell carcinoma (KEYNOTE-040): a randomised, open-label, phase 3 study. *Lancet.* (2019) 393:156–167. doi: 10.1016/S0140-6736(18)31999-8.
8. Chen R, Zinzani PL, Fanale MA, Armand P, Johnson NA, Brice P, et al; KEYNOTE-087. Phase II study of the efficacy and safety of pembrolizumab for relapsed/refractory classic hodgkin lymphoma. *J Clin Oncol.* (2017) 35:2125–2132. doi: 10.1200/JCO.2016.72.1316.
9. Zinzani PL, Ribrag V, Moskowitz CH, Michot JM, Kuruvilla J, Balakumaran A, et al. Safety and tolerability of pembrolizumab in patients with relapsed/refractory primary mediastinal large B-cell lymphoma. *Blood.* (2017) 130:267-270. doi: 10.1182/blood-2016-12-758383.
10. Balar AV, Castellano D, O'Donnell PH, Grivas P, Vuky J, Powles T, et al. First-line pembrolizumab in cisplatin-ineligible patients with locally advanced and unresectable or metastatic urothelial cancer (KEYNOTE-052): a multicentre, single-arm, phase 2 study. *Lancet Oncol.* (2017) 18:1483–1492. doi: 10.1016/S1470-2045(17)30616-2.
11. Bellmunt J, de Wit R, Vaughn DJ, Fradet Y, Lee JL, Fong L, et al; KEYNOTE-045 Investigators. Pembrolizumab as second-line therapy for advanced urothelial carcinoma. *N Engl J Med*. (2017) 376:1015–1026. doi: 10.1056/NEJMoa1613683.
12. Chang L, Chang M, Chang HM, Chang F. Microsatellite instability: A predictive biomarker for cancer immunotherapy. *Appl Immunohistochem Mol Morphol.* (2018) 26:e15–e21. doi: 10.1097/PAI.0000000000000575.
13. Smyth EC, Petty RD. Pembrolizumab versus paclitaxel in gastro-oesophageal adenocarcinoma. *Lancet.* (2018) 392:97–98. doi: 10.1016/S0140-6736(18)31277-7.
14. Shitara K, Özgüroğlu M, Bang YJ, Di Bartolomeo M, Mandalà M, Ryu MH, et al; KEYNOTE-061 investigators. Pembrolizumab versus paclitaxel for previously treated, advanced gastric or gastro-oesophageal junction cancer (KEYNOTE-061): a randomised, open-label, controlled, phase 3 trial. *Lancet.* (2018) 392:123–133. doi: 10.1016/S0140-6736(18)31257-1.
15. Frenel JS, Le Tourneau C, O'Neil B, Ott PA, Piha-Paul SA, Gomez-Roca C, et al. Safety and efficacy of pembrolizumab in advanced, programmed death ligand 1-positive cervical cancer: results from the Phase Ib KEYNOTE-028 Trial. *J Clin Oncol.* (2017) 35:4035–4041. doi: 10.1200/JCO.2017.74.5471.
16. Zhu AX, Finn RS, Edeline J, Cattan S, Ogasawara S, Palmer D, et al; KEYNOTE-224 investigators. Pembrolizumab in patients with advanced hepatocellular carcinoma previously treated with sorafenib (KEYNOTE-224): a non-randomised, open-label phase 2 trial. *Lancet Oncol.* (2018) 19:940–952. doi: 10.1016/S1470-2045(18)30351-6.
17. Nghiem P, Bhatia S, Lipson EJ, Sharfman WH, Kudchadkar RR, Brohl AS, et al. Durable tumor regression and overall survival in patients with advanced merkel cell carcinoma receiving pembrolizumab as first-line therapy. *J Clin Oncol.* (2019) 37:693–702. JCO1801896. doi: 10.1200/JCO.18.01896.
18. Hazarika M, Chuk MK, Theoret MR, Mushti S, He K, Weis SL, et al. U.S. FDA Approval Summary: nivolumab for treatment of unresectable or metastatic melanoma following progression on ipilimumab. *Clin Cancer Res*. (2017) 23:3484–3488. doi: 10.1158/1078-0432.CCR-16-0712.
19. Hodi FS, Chesney J, Pavlick AC, Robert C, Grossmann KF, McDermott DF, et al. Combined nivolumab and ipilimumab versus ipilimumab alone in patients with advanced melanoma: 2-year overall survival outcomes in a multicentre, randomised, controlled, phase 2 trial. *Lancet Oncol.* (2016) 17:1558–1568. doi: 10.1016/S1470-2045(16)30366-7.
20. Larkin J, Chiarion-Sileni V, Gonzalez R, Grob JJ, Cowey CL, Lao CD, et al. Combined nivolumab and ipilimumab or monotherapy in untreated melanoma. *N Engl J Med.* (2015) 373:23–34. doi: 10.1056/NEJMoa1504030.
21. Weber J, Mandala M, Del Vecchio M, Gogas HJ, Arance AM, Cowey CL, et al; CheckMate 238 Collaborators. Adjuvant nivolumab versus ipilimumab in resected stage III or IV melanoma. *N Engl J Med.* (2017) 377:1824–1835. doi: 10.1056/NEJMoa1709030.
22. Horn L, Spigel DR, Vokes EE, Holgado E, Ready N, Steins M, et al. Nivolumab versus docetaxel in previously treated patients with advanced non-small-cell lung cancer: two-year outcomes from two randomized, open-label, phase III trials (CheckMate 017 and CheckMate 057). *J Clin Oncol.* (2017) 35:3924–3933. doi: 10.1200/JCO.2017.74.3062.
23. Borghaei H, Paz-Ares L, Horn L, Spigel DR, Steins M, Ready NE, et al. Nivolumab versus docetaxel in advanced nonsquamous non-small-cell lung cancer. *N Engl J Med*. (2015) 373:1627–1639. doi: 10.1056/NEJMoa1507643.
24. Brahmer J, Reckamp KL, Baas P, Crinò L, Eberhardt WE, Poddubskaya E, et al. Nivolumab versus docetaxel in advanced squamous-cell non-small-cell lung cancer. *N Engl J Med*. (2015) 373:123–135. doi: 10.1056/NEJMoa1504627.
25. Antonia SJ, López-Martin JA, Bendell J, Ott PA, Taylor M, Eder JP, et al. Nivolumab alone and nivolumab plus ipilimumab in recurrent small-cell lung cancer (CheckMate 032): a multicentre, open-label, phase 1/2 trial. *Lancet Oncol.* (2016) 17:883–895. doi: 10.1016/S1470-2045(16)30098-5.
26. Motzer RJ, Tannir NM, McDermott DF, Arén Frontera O, Melichar B, Choueiri TK, et al; CheckMate 214 Investigators. Nivolumab plus ipilimumab versus sunitinib in advanced renal-cell carcinoma. *N Engl J Med.* (2018) 378:1277–1290. doi: 10.1056/NEJMoa1712126.
27. Motzer RJ, Escudier B, McDermott DF, George S, Hammers HJ, Srinivas S, et al; CheckMate 025 Investigators. Nivolumab versus everolimus in advanced renal-cell carcinoma. *N Engl J Med*. (2015) 373:1803–1813. doi: 10.1056/NEJMoa151066.
28. Younes A, Santoro A, Shipp M, Zinzani PL, Timmerman JM, Ansell S, et al. Nivolumab for classical Hodgkin's lymphoma after failure of both autologous stem-cell transplantation and brentuximab vedotin: a multicentre, multicohort, single-arm phase 2 trial. *Lancet Oncol.* (2016) 17:1283–1294. doi: 10.1016/S1470-2045(16)30167-X.
29. Ansell SM, Lesokhin AM, Borrello I, Halwani A, Scott EC, Gutierrez M, et al. PD-1 blockade with nivolumab in relapsed or refractory Hodgkin's lymphoma. *N Engl J Med*. (2015) 372:311–319. doi: 10.1056/NEJMoa1411087.
30. Ferris RL, Blumenschein G Jr, Fayette J, Guigay J, Colevas AD, Licitra L, et al. Nivolumab for recurrent squamous-cell carcinoma of the head and neck. *N Engl J Med*. (2016) 375:1856–1867.
31. Sharma P, Callahan MK, Bono P, Kim J, Spiliopoulou P, Calvo E, et al. Nivolumab monotherapy in recurrent metastatic urothelial carcinoma (CheckMate 032): a multicentre, open-label, two-stage, multi-arm, phase 1/2 trial. *Lancet Oncol.* (2016) 17:1590–1598. doi: 10.1016/S1470-2045(16)30496-X.
32. Overman MJ, McDermott R, Leach JL, Lonardi S, Lenz HJ, Morse MA, et al. Nivolumab in patients with metastatic DNA mismatch repair-deficient or microsatellite instability-high colorectal cancer (CheckMate 142): an open-label, multicentre, phase 2 study. *Lancet Oncol.* (2017) 18:1182–1191. doi: 10.1016/S1470-2045(17)30422-9. Erratum in: [*Lancet Oncol.* (2017) 18:e510](https://www.ncbi.nlm.nih.gov/pubmed/28884691).
33. Overman MJ, Lonardi S, Wong KYM, Lenz HJ, Gelsomino F, Aglietta M, et al. Durable clinical benefit with nivolumab plus ipilimumab in DNA mismatch repair-deficient/microsatellite instability-high metastatic colorectal cancer. *J Clin Oncol.* (2018) 36:773–779. doi: 10.1200/JCO.2017.76.9901.
34. Feng D, Hui X, Shi-Chun L, Yan-Hua B, Li C, Xiao-Hui L, et al. Initial experience of anti-PD1 therapy with nivolumab in advanced hepatocellular carcinoma. *Oncotarget*. (2017) 8:96649–96655. doi: 10.18632/oncotarget.20029.
35. Migden MR, Rischin D, Schmults CD, Guminski A, Hauschild A, Lewis KD, et al. PD-1 blockade with cemiplimab in advanced cutaneous squamous-cell carcinoma. *N Engl J Med.* (2018) 379(4):341–351. doi: 10.1056/NEJMoa1805131.
36. Powles T, Durán I, van der Heijden MS, Loriot Y, Vogelzang NJ, De Giorgi U, et al. Atezolizumab versus chemotherapy in patients with platinum-treated locally advanced or metastatic urothelial carcinoma (IMvigor211): a multicentre, open-label, phase 3 randomised controlled trial. *Lancet.* (2018) 391:748–757. doi: 10.1016/S0140-6736(17)33297-X. Erratum in: [*Lancet.* (2018) 392:1402](https://www.ncbi.nlm.nih.gov/pubmed/30343855).
37. Rittmeyer A, Barlesi F, Waterkamp D, Park K, Ciardiello F, von Pawel J, et al; OAK Study Group. Atezolizumab versus docetaxel in patients with previously treated non-small-cell lung cancer (OAK): a phase 3, open-label, multicentre randomised controlled trial. *Lancet*. (2017) 389:255–265. doi: 10.1016/S0140-6736(16)32517-X.
38. Powles T, O'Donnell PH, Massard C, Arkenau HT, Friedlander TW, Hoimes CJ, et al. Efficacy and safety of durvalumab in locally advanced or metastatic urothelial carcinoma: updated results from a Phase 1/2 open-label study. *JAMA Oncol.* (2017) 3:e172411. doi: 10.1001/jamaoncol.2017.2411.
39. Antonia SJ, Villegas A, Daniel D, Vicente D, Murakami S, Hui R, et al; PACIFIC Investigators. Overall survival with durvalumab after chemoradiotherapy in Stage III NSCLC. *N Engl J Med.* (2018) 379:2342–2350. doi: 10.1056/NEJMoa1809697.
40. Kaufman HL, Russell J, Hamid O, Bhatia S, Terheyden P, D'Angelo SP, et al. Avelumab in patients with chemotherapy-refractory metastatic Merkel cell carcinoma: a multicentre, single-group, open-label, phase 2 trial. *Lancet Oncol*. (2016) 17:1374–1385. doi: 10.1016/S1470-2045(16)30364-3.
41. Weber J, Mandala M, Del Vecchio M, Gogas HJ, Arance AM, Cowey CL, et al; CheckMate 238 Collaborators. Adjuvant nivolumab versus ipilimumab in resected Stage III or IV melanoma. *N Engl J Med.* (2017) 377:1824–1835. doi: 10.1056/NEJMoa1709030.
